# Accuracies of Genomic Prediction for Growth Traits at Weaning and Yearling Ages in Yak

**DOI:** 10.3390/ani10101793

**Published:** 2020-10-02

**Authors:** Fei Ge, Congjun Jia, Pengjia Bao, Xiaoyun Wu, Chunnian Liang, Ping Yan

**Affiliations:** Key Laboratory of Yak Breeding Engineering of Gansu Province, Lanzhou Institute of Husbandry and Pharmaceutical Sciences, Chinese Academy of Agricultural Sciences, Lanzhou 730050, China; 82101185176@caas.cn (F.G.); dkjcj@nwafu.edu.cn (C.J.); baopengjia@caas.cn (P.B.); wuxiaoyun@caas.cn (X.W.)

**Keywords:** genomic prediction, yak, growth traits, GBLUP, Bayesian approaches, genomic estimation of breeding value (GEBV)

## Abstract

**Simple Summary:**

Genomic selection is a new technology in animal breeding after the selection according to the best linear unbiased prediction (BLUP) value and marker assisted selection. Genomic selection has gradually been used in practical applications over recent years following the advent of high-density single nucleotide polymorphism (SNP) chips for livestock and poultry. Yak are a critical species on the Qinghai–Tibet Plateau, which is of great significance to herders. The early selection of yak could save feeding costs and shorten the generation interval. In the present study, we estimated the accuracy of genomic prediction compared with different classical models for yak early growth traits. The results of cross-validation indicated that the average predictive accuracy ranged from 0.147 to 0.391. The average correlation coefficient between prediction and true phenotype was 0.4.

**Abstract:**

Genomic selection is a promising breeding strategy that has been used in considerable numbers of breeding projects due to its highly accurate results. Yak are rare mammals that are remarkable because of their ability to survive in the extreme and harsh conditions predominantly at the so-called “roof of the world”—the Qinghai–Tibetan Plateau. In the current study, we conducted an exploration of the feasibility of genomic evaluation and compared the predictive accuracy of early growth traits with five different approaches. In total, four growth traits were measured in 354 yaks, including body weight, withers height, body length, and chest girth in two early stages of development (weaning and yearling). Genotyping was implemented using the Illumina BovineHD BeadChip. The predictive accuracy was calculated through five-fold cross-validation in five classical statistical methods including genomic best linear unbiased prediction (GBLUP) and four Bayesian methods. Body weights at 30 months in the same yak population were also measured to evaluate the prediction at 6 months. The results indicated that the predictive accuracy for the early growth traits of yak ranged from 0.147 to 0.391. Similar performance was found for the GBLUP and Bayesian methods for most growth traits. Among the Bayesian methods, Bayes B outperformed Bayes A in the majority of traits. The average correlation coefficient between the prediction at 6 months using different methods and observations at 30 months was 0.4. These results indicate that genomic prediction is feasible for early growth traits in yak. Considering that genomic selection is necessary in yak breeding projects, the present study provides promising reference for future applications.

## 1. Introduction

Animal breeding has been traditionally conducted through conventional phenotypic selection followed by the evaluation of estimated breeding values (EBVs), using best linear unbiased prediction (BLUP) [1]. In the wake of developments in high throughput sequencing and chip technology, genomic prediction was proposed in 2001, as genomic estimated breeding values (GEBVs), inferred from dense genetic markers, were more accurate than traditional EBVs based on pedigree [2,3]. Thus, candidate animals can be selected from their GEBV ranking in advance. The theoretical assumption of genomic prediction is that each quantitative trait locus (QTL) which may affect the target trait may be in linkage disequilibrium (LD) with at least one genetic marker, such as a single nucleotide polymorphism (SNP), throughout the genome. Compared with pedigree-based EBV, there are some advantages for which genomic selection is more desirable in modern animal breeding programs. It has been reported that genomic prediction could integrate the differences in genetic information between individuals and separate actual genetic effects from phenotypic variation [2]. In addition, the genomic predictive model could capture small effects and simultaneously fit all markers in an unbiased estimation, eliminating the need for the testing of progeny. The increased genetic progress and lower breeding costs brought about by genomic prediction could also solve the restrictions of traditional patterns of breeding [4]. In general, genomic selection comprises three phases: (i) Establishment of a reference population for which phenotypic and genotypic data are known; (ii) Estimation of the GEBVs of candidate individuals (for which the genotypes are available). Direct approaches derive GEBVs through the construction of a marker-derived relationship matrix. Indirect approaches integrate the effect of each marker to calculate a GEBV. (iii) Selection of animals with target traits, based on their GEBV ranking.

The use of genomic selection in dairy cattle breeding has been widely implemented in many countries, such as the USA [5], Australia [6], Norway [7], New Zealand [8] and France [9]. It has been reported that if genomic prediction were used, a ~50–100% increase in annual genetic gain rates would be obtained in US Holstein cattle [10]. Substantial efforts in genomic evaluation have now been conducted in many breeding projects in farm animal species. The predictive accuracy of GS is represented by the correlation coefficient of GEBV with true breeding values, and ranges from zero to one [2]. For example, Piccoli et al. calculated the predictive accuracy of GEBVs across different scenarios in beef cattle, ranging from 0.38 to 0.40 for approximately twenty economic traits [11]. As reported by Brito et al., the predictive accuracy of growth traits in New Zealand sheep breeds ranged from 0.18 to 0.33 [12]. In addition, studies from PIC, a pig breeding company, indicated that the predictive accuracy of GEBV was 0.42, 0.50, 0.50, and 0.51 for the following beneficial traits: number of piglets in a litter, growth rate, feed intake, and loin depth, respectively [13]. Attempts at genomic selection have also been implemented in bird species and aquatic animals [14,15].

The yak, an indispensable but rare member of the Bovidae family living on the Qinghai–Tibet Plateau, supplies meat, milk, transportation, and fur to local herdsmen. The Asidan yak is a new variety bred by Chinese scientists, characterized by its notably docile temperament and outstanding performance. Increasingly, the mode of grazing in yak is changing into intensive farming, along with a transformation of the lifestyle of herdsmen. Therefore, it is desirable to apply genomic selection to the yak population in order to facilitate gains in the overall level of production. To take full advantage of genomic selection, we have placed emphasis on selection for early growth traits compared to a previous study concerning the preliminary exploration of genomic prediction in yak [16]. Furthermore, genomic selection combined with early growth traits could lead to considerably decreased feeding costs, as genotyped animals could be procured when juvenile. Thus, our objective was to evaluate the feasibility of applying genomic prediction in the yak population by assessing values of predictive accuracy. Here, we compared the predictive accuracy of early growth traits (body weight, withers height, body length, and chest girth) using five approaches at two different ages (weaning and yearling).

## 2. Materials and Methods

### 2.1. Ethics Statement

The procedures for yak blood sample collection and body measurements were consistent with the guidelines approved by the Lanzhou Institute of Husbandry and Pharmaceutical Sciences (No. LIHPS-CAAS-2017-115).

### 2.2. Populations and Phenotypes

A total of 354 female Ashidan yaks were randomly selected from the Datong yak farm located in Datong county, Qinghai province, China. All animals were born between March and May 2017 and grazed freely on natural pasture with ad libitum drinking water.

Yak growth traits were measured at weaning (6 months), yearling (12 months) and adult (30 months), respectively. A total of four traits were examined and analyzed: body weight (BW; kg), withers height (WH; cm), body length (BL; cm), and chest girth (CG; cm). The summary statistics of animal phenotypes are presented in Table 1.

### 2.3. Genotypes and Quality Control

**Figure 1:**
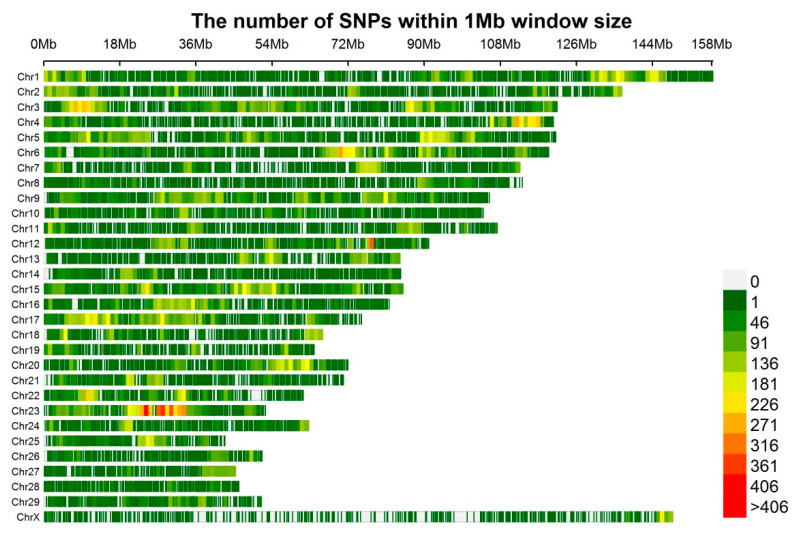
Density of single nucleotide polymorphisms (SNPs) on each chromosome after quality control.

Blood was sampled from the jugular vein for genomic DNA extraction. An EasyPure Blood Genomic DNA kit (TransGen Biotech, Co., Ltd., Beijing, China) was used to extract genomic DNA. Quantified DNA samples were stored at −20 °C. All animals were genotyped using an Illumina BovineHD BeadChip (Illumina Inc., San Diego, CA, USA). A total of 777,962 variants were included in this chip at an average spacing of 3.43 kb. The SNPs with call rate ≤ 0.90, *p*-values < 10^−6^ for the Hardy–Weinberg Equilibrium test and minor allele frequency < 0.01 were removed before subsequent analysis. Individuals were retained where the genotype call rate >0.90. Plink v1.90 software (http://www.cog-genomics.org/plink2) was used for the quality control of data. A total of 642,021 variants were removed due to failing to pass the minor allele threshold. The distribution of minor allele frequency of SNPs after quality control is displayed in Figure S1. The MAF distribution and density of SNPs of each chromosome are shown in Table S1 and Figure 1. After the data quality control exercise, a total of 98,688 variants from 354 animals were used in subsequent analyses. The rMVP software (Wuhan, China) was used to create the marker density figure [17]. The number of SNPs within a 1 Mb window on each chromosome is shown in Figure 1.

### 2.4. Statistical Models

Variance component estimation and heritability calculations were performed using the restricted maximum likelihood method using BLUPF90 software [18]. A relationship matrix was constructed using SNP data. Trait heritability (*h^2^*) was calculated by dividing additive genetic variance by total variance, as follows:

(1) $$h^{2}\;=\;\frac{\sigma_{a}^{2}}{\sigma_{a}^{2}+\sigma_{e}^{2}}$$

where $\sigma_{a}^{2}$ represented additive genetic variance, and $\sigma_{e}^{2}$ denoted environmental variance.

The accuracy of the predictive capability of five approaches, including genomic best linear unbiased prediction (GBLUP) and four Bayesian methods was compared. Furthermore, only additive effects, and not fixed effects, were considered in the present study.

#### 2.4.1. GBLUP

For the calculation of GBLUP, a **G** matrix was substituted for the traditional **A** matrix normally constructed using a pedigree. The **G** matrix—constructed using SNP data—was used for estimating the relationships between animals [19]. The statistical model is presented as:

(2) y=μ1+Zg+e

where ***y***, μ, ***Z***, ***g***, and ***e*** denote the phenotype vector, the overall constant, incidence matrix that links ***g***, the GEBV vector, and residual vector, respectively. It was assumed that $g_{j}$ was represented by the normal distribution N (0, G$\sigma_{g}^{2}$), where $\sigma_{g}^{2}$ denotes additive genetic variance. The residual vector ***e*** was represented by the normal distribution ***e***~N (**0**, $I\sigma_{e}^{2}$).

#### 2.4.2. Bayes A, Bayes B, Bayes C, and Bayes Lasso

Distinct from GBLUP, Bayesian methods assume that the variance structure explained by each locus is unequal. The statistical model in Bayesian methods is based on the following:

(3) $$y_{i}=\mu +\Sigma_{j=1}^{n}\left(z_{i_{j}}g_{j}\right)+e_{i}$$

where $y_{i}$ represents the phenotype vector of individual *i*, μ represents the overall constant, $z_{i_{j}}$ denotes the genotype indicator vector of marker *j* for individual *i*, *n* represents the number of markers, $g_{j}$ represents the vector for the effects of markers and $e_{i}$ denotes random errors represented by the normal distribution N (0, $I\sigma_{e}^{2}$). The value of $z_{i_{j}}$ is 0, 1 or 2, corresponding to homozygous, heterozygous, or another homozygous genotype.

In Bayes A, every SNP is assumed to have an effect, with the posterior distribution following a normal distribution $g_{j}\sim N(0,\;\sigma_{g_{j}}^{2}$), where the variance $\sigma_{g_{j}}^{2}$ is represented by the inverse chi-square distribution $\sigma_{g_{j}}^{2}$~$\chi^{-2}$(ν,S) in which ν denotes the number of degrees of freedom and S is a scale parameter.

The Bayes B and Bayes C methods assume that the majority of SNPs contribute no effect while a minority does. The proportion of SNPs having an effect was deemed to be 1 − π [20]. The prior distribution of π in Bayes B was a constant while π~uniform (0, 1) in Bayes C. The posterior distribution and effect variance distribution in Bayes B and Bayes C were the same as that described for Bayes A, namely $g_{j}\sim N(0,\;\sigma_{g_{j}}^{2}$) and $\sigma_{g_{j}}^{2}$~$\chi^{-2}$(ν,S).

Bayesian Lasso regression is similar to Bayes A, except that the SNP effect variance complies with the Laplace distribution $\sigma_{g_{j}}^{2}$~Exp ($\lambda^{2}$/2) [21]. For the conjugate, the prior distribution of parameter λ is $p\left(\lambda^{2}|a,b\right)=Gamma\;(\lambda^{2}|a,b)$.

The Bayesian formulae can thus be represented as follows:

(4) $$\left\{\begin{array}{c} Bayes\;A:a\;priori\;hypothesis:g_{i}=0,posterior\;hypothesis:g_{i}\sim N\left(0,\sigma_{g}^{2}\right),\;variance\;hypothesis:\sigma_{g}^{2}\sim \chi^{-2}\left(\nu ,S\right)\\ Bayes\;B:a\;priori\;hypothesis:g_{i}=constant,\;posterior\;hypothesis:g_{i}\sim N\left(0,\sigma_{g}^{2}\right),\;variance\;hypothesis:\sigma_{g}^{2}\sim \chi^{-2}\left(\nu ,S\right)\\ Bayes\;C:a\;priori\;hypothesis:g_{i}=U\left(0,1\right),\;posterior\;hypothesis:g_{i}\sim N\left(0,\sigma_{g}^{2}\right),\;variance\;hypothesis:\sigma_{g}^{2}\sim \chi^{-2}\left(\nu ,S\right)\\ Bayes\;Lasso:a\;priori\;hypothesis:g_{i}=0,posterior\;hypothesis:g_{i}\sim N\left(0,\sigma_{g}^{2}\right),variance\;hypotheis:\sigma_{g}^{2}\sim Exp\left(\frac{\lambda^{2}}{2}\right) \end{array}\right\}$$

BLUPF90 software (Athens, GA, USA) was used to calculate GBLUP [18]. Bayesian methods analysis was conducted using the BGLR package (Birmingham, AL, USA) in the R programming environment [22].

### 2.5. Evaluation of Genomic Prediction Accuracy

Five-fold cross-validation was implemented to assess the accuracy of the predictions. The 354 individual animals were randomly allocated into five groups, of which four groups had genotypes and phenotypes considered to be the reference population whereas phenotypes in the validation population were recorded as unknown. Predictive ability was calculated as the correlation between phenotype and GEBV. Additionally, to avoid any influence of heritability, the following formula was used to evaluate the accuracy of prediction [23]:

(5) $$r=\frac{Cor\left(GEBV,y\right)}{\sqrt{h^{2}}}$$

where *y* was the observed phenotype, and $h^{2}$ denoted heritability. The training and validation populations were randomly sampled and the program repeated ten times. The mean of the correlation values was considered a measure of predictive accuracy.

For further testing and verifying the feasibility of genomic prediction, a Pearson correlation test was performed between phenotypes estimated at BW6 with different approaches and observations at BW30. Analyses were conducted using IBM SPSS Statistics software (Version 23, IBM, New York, NY, USA).

## 3. Results

### 3.1. Estimation of Genetic Parameters

As shown in Table 2, heritability ranged from 0.07 (BL12) to 0.57 (WH6). Of these, BL6 (0.56) and WH6 (0.57) exhibited high heritability, whereas the majority of traits displayed moderate heritability: CG6 (0.39), BW6 (0.35), CG12 (0.25), BW12 (0.24) and WH12 (0.22). BL12 (0.07) exhibited low heritability. Interestingly, heritability of the same trait declined across the age groups, especially when comparing heritability of body length at 6 and 12 months. We have discussed the possible reasons later in this article. Details of the genetic parameters are displayed in Table 2.

### 3.2. Predictive Ability and Accuracy

**Figure 2:**
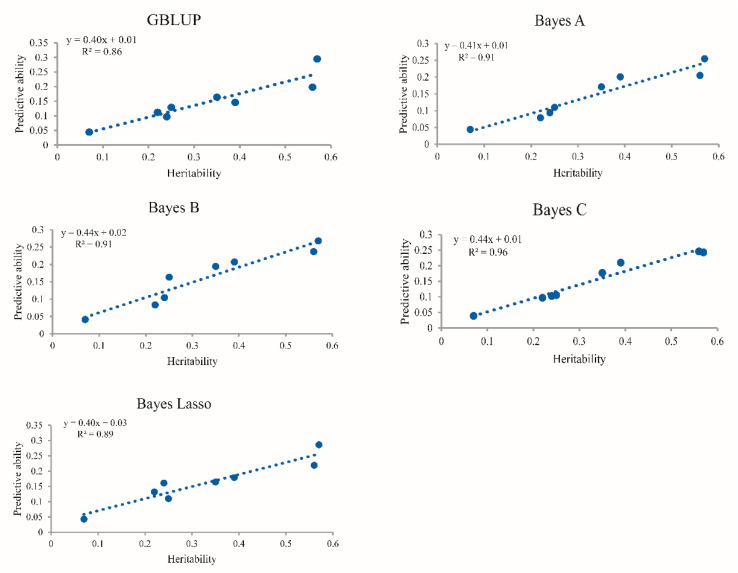
Regression of predictive ability on heritability for early growth traits using five approaches. GBLUP: genomic best linear unbiased prediction.

Table 3 presents the predictive ability and accuracy of the five statistical methods based on five-fold cross-validation. The predictive ability ranged from 0.039 (BL12 in Bayes C) to 0.295 (WH6 in GBLUP). It is worth noting that traits with low heritability tended to have inferior predictive ability compared to traits with higher heritability. The regression coefficient was calculated in order to explore the affiliation between heritability and predictive ability (Figure 2). The corresponding regression coefficients for the five methods were 0.40 (GBLUP), 0.41 (Bayes A), 0.44 (Bayes B and Bayes C), and 0.40 (Bayes Lasso).

For predictive accuracy, the means were 0.253, 0.246, 0.275, 0.258, and 0.278 corresponding to GBLUP, Bayes A, Bayes B, Bayes C, and Bayes Lasso methods for different traits, respectively. The integral of predictive accuracy ranged from 0.147 to 0.391. It should be noted that the predictive accuracy of GBLUP was similar to that of the Bayesian methods. In particular, Bayes B outperformed Bayes A for the majority of traits. Additionally, GBLUP exhibited the worst performance for BW6, BL6 and CG6. Conversely, GBLUP displayed the greatest accuracy for WH6 and BL12 in comparison with the Bayesian methods.

### 3.3. Correlation between Predicted Phenotype and Observation

The correlation coefficient for the prediction using different models at BW6 and observations at BW30 are presented in Table 4. Overall, the mean correlation coefficient of the five statistical models was 0.402 all of which were highly significant, indicating that the genomic prediction of body weight was accurate.

## 4. Discussion

The superiority of genomic selection represents an acceleration of genetic progression and a shortening of the generation interval compared with traditional breeding. Growth traits, as a representation of important economic traits, are key indicators in the breeding programs of livestock. In a previous study, Saatchi et al. reported that the accuracy of genomic prediction for weaning and yearling weight was 0.33 and 0.36 in Angus cattle [24]. It has been demonstrated in studies of Chinese Simmental beef cattle that the predictive accuracy of growth traits was 0.312 and 0.398, corresponding to mean daily weight gain and live weight, respectively [25]. However, a higher predictive accuracy, of 0.52–0.56, was observed in growth traits in US beef cattle [26]. The present study estimated the accuracy of genomic prediction of early growth traits based on five classical methods using a real Ashidan yak dataset. Genotyping was performed using the commercialized BovineHD BeadChip (Illumina Inc., San Diego, CA, USA). There is no customized chip for Yak as yet because the sequencing costs are relatively high. As shown in the BovineHD BeadChip manual, yak is covered in SNPs validation as an outgroup species. The applicability of this chip for yak has also been verified in a study with regard to the introgressive hybridization in yaks [27]. Although most variants are poorly polymorphic in yak, the distribution of variants is relatively uniform on each chromosome after quality control (Table S1). As shown in Figure S1, the minor allele frequency of the SNPs is approximately uniformly distributed, starting from 0.06. The results of variants quality control indicate that the follow-up genomic prediction can be conducted.

### 4.1. Comparison of Approaches to Genomic Prediction

The particular statistical model used in genomic prediction has been demonstrated to be critical in the identification of superior livestock [28]. Direct estimation methods, such as GBLUP, consider individual animals as random effects and estimate the components of variance using an iterative method, subsequently solving mixed model equations to obtain a GEBV of candidate individuals. Conversely, indirect methods, such as Bayesian methods, evaluate the effects of SNPs in a training population, then accumulate the effects to obtain the GEBV of a candidate population. In the present study, we calculated the predictive accuracy in four early growth traits with different approaches, e.g., WH6 using GBLUP (0.391) and Bayes Lasso (0.379). Furthermore, we found that Bayes B displayed greater accuracy than Bayes A for the majority of traits. A possible reason may be due to the a priori hypothesis of Bayes B, that better captures Mendelian sampling and which is closer to genetic architecture of economic traits. Bayes B and Bayes Lasso exhibited similar accuracy in the present study. So far, no consensus statistical approach has been shown to apply to all traits.

A number of studies that have compared the accuracy of GBLUP and Bayesian methods have been previously reported. Sun et al., using simulated data, reported that Bayesian regression approaches are superior to GBLUP [29]. Similar results have been demonstrated in the Nordic Holstein population [30]. In addition, studies using a simulated dataset from the 13th QTL-MAS Workshop implied that the Bayes Lasso method yielded greater accuracy than Bayes A [31]. Nonetheless, it has been reported that GBLUP outperformed Bayes B for carcass merit traits in 543 Angus and 400 Charolais beef cattle [32]. Additionally, Hayes et al. reported that GBLUP performed with similar accuracy to Bayes B in a multibreed dairy population [3]. Evidence has shown that Bayesian methods outperform GBLUP when considering traits in which large effects are generated by only a few QTLs (quantitative trait loci) [5,33]. Considering that the majority of growth traits are controlled by a few QTLs with large effects and multiple genes with limited effects, a similar predictive accuracy was observed using GBLUP and Bayesian methods in the present study. This is not the only case where this has been observed. Studies in plant breeding have reported that methods with conceptual differences present a very similar predictive accuracy [34].

### 4.2. Impact of Heritability on the Accuracy of Genomic Prediction

Heritability is defined as the proportion of genetic variance or additive genetic variance that contributes to the variance of phenotype. Studies have shown that traits with higher heritability often display considerably more genomic predictive accuracy than those traits with lower heritability [35,36,37]. As expected, this is in accordance with our findings. WH6, which had the highest heritability yielded higher accuracy when GBLUP and Bayes Lasso were used. BL12 had the lowest heritability in the present study and also displayed the lowest predictive accuracy compared with other traits. Therefore, we further investigated the correlation between trait heritability and predictive capability, for which positive linear correlations were observed. Interestingly, when we compared the same trait across different age groups, a decreasing trend was found in heritability from 6 to 12 months. This phenomenon may be due to the phenotype of growth traits becoming relatively uniform at yearling ages. There are some reasons for the changes in phenotypic variation from weaning to yearling. Firstly, yak calves with dams of higher lactation capability may suffer severe weanling stress in the cold season because of their poor herbivorous capability, which might result in a disturbance to their growth. Carves of dams with poor milking performance, however, which are more successful herbivores would pass the transition stably. Thus, the records of body composition traits as well as body weight at yearling are more uniform than those at weaning. Secondly, calf yaks have to endure an extremely cold season with an insufficient supply of hay for six months after weaning. The severe shortage of forage may lead to an unstable phenotype. The heritability calculated for BL12 was 0.07 which is abnormally lower than other traits. One possible explanation which we speculate could be related to the measure of deviation. Yak that fear humans are prone to bow at the waist as preadaptive behavior when measuring conformational traits such as body length.

In addition, we primarily focused on the trait of body weight, considered a major indicator of production in the yak. Correlation analysis was conducted between the prediction of BW6 and observations at BW30. The results verify that reliable genomic evaluation is feasible and necessary in the early period in yak. Weaning and yearling weights embody the growing potential of livestock and represent common criteria for the assessment of bulls in beef cattle in many countries. There are additional challenges for the implementation of genomic selection in yak due to environmental constraints and outdated breeding technology. Nevertheless, with the attention of grassland ecological conservation, intensive feeding will progressively substitute for smallholder farming. Not only that, the government have created policies that provide governance and support for the industrialization of yak which is a pillar industry in Tibet. The difficulty in data collection will improve little-by-little. In this regard, considering that the yak is an essential species for herdsmen on the plateau, we performed genomic prediction using a number of basic methods in a limited population size and discussed the factors that may influence its predictive accuracy. The results prove that genomic prediction can be exploited in yak selection programs.

## 5. Conclusions

The feasibility of genomic prediction of early growth traits in the yak population was explored in the present study. To estimate breeding values, five different approaches were conducted and individually compared in 354 Ashidan yaks for four traits in weaning and yearling animals. The results indicate the accuracy of prediction ranged from 0.147 to 0.391. For the majority of traits, the accuracy of Bayesian methods was similar to that of GBLUP. However, additional investigation, using alternative predictive approaches, with a greater marker density, and/or a larger training population size might be warranted in the future for yak genomic selection.

## Figures and Tables

**Table 1:** Summary and definition of phenotypic traits, numbers of animals, mean values, standard deviations (SD), and minimum and maximum values.

Trait	Number	Mean	SD	Minimum	Maximum	Trait Definition
BW6	350	84.18	10.31	58	117	Body weight at 6 months, kg
WH6	354	94.37	5.26	82	108	Withers height at 6 months, cm
BL6	354	91.89	7.379	73	116	Body length at 6 months, cm
CG6	354	124.03	7.809	100	144	Chest girth at 6 months, cm
BW12	343	82.57	10.51	48	113	Body weight at 12 months, kg
WH12	349	90.49	4.18	81	102	Withers height at 12 months, cm
BL12	349	95.93	4.957	80	113	Body length at 12 months, cm
CG12	349	117.16	5.08	102	134	Chest girth at 12 months, cm
BW30	263	155.42	15.23	108	203	Body weight at 30 months, kg
WH30	267	99.55	4.997	90	117	Withers height at 30 months, cm
BL30	265	113.17	5.696	96	126	Body length at 30 months, cm
CG30	261	146.97	8.266	122	173	Chest girth at 30 months, cm

Abbreviations: body weight (BW; kg), withers height (WH; cm), body length (BL; cm), and chest girth (CG; cm).

**Table 2:** Variance component estimation and the heritability of four traits for two stages of development.

Trait	$\sigma_{a}^{2}$	$\sigma_{e}^{2}$	$h^{2}$
BW6	37.506	68.848	0.35 ± 0.01
WH6	15.597	11.558	0.57 ± 0.02
BL6	30.752	24.181	0.56 ± 0.02
CG6	23.822	36.526	0.39 ± 0.01
BW12	26.815	83.298	0.24 ± 0.01
WH12	3.941	13.511	0.22 ± 0.02
BL12	1.746	22.812	0.07 ± 0.04
CG12	6.493	19.171	0.25 ± 0.02

$\sigma_{a}^{2}$ represents additive genetic variance; $\sigma_{e}^{2}$ represents environmental variance; $h^{2}$ represents heritability; BW6: body weight at 6 months; WH6: withers height at 6 months; BL6: body length at 6 months; CG6: chest girth at 6 months, BW12: body weight at 12 months; WH12: withers height at 12 months; BL12: body length at 12 months; CG12: chest girth at 12 months.

**Table 3:** Predictive ability and accuracy of early growth traits for two developmental stages using five statistical methods with five-fold cross-validation.

Trait	$\mathrm{Predictive}\;\mathrm{Ability}\;r_{GEBV,y}$					$\mathrm{Predictive}\;\mathrm{Accuracy}\;r_{GEBV,y}/\sqrt{h^{2}}$				
	GBLUP	BayesA	BayesB	BayesC	Bayes Lasso	GBLUP	BayesA	BayesB	BayesC	Bayes Lasso
BW6	0.164	0.171	0.194	0.177	0.164	0.277	0.289	0.328	0.299	0.277
WH6	0.295	0.255	0.268	0.243	0.286	0.391	0.338	0.355	0.322	0.379
BL6	0.198	0.205	0.237	0.246	0.219	0.265	0.274	0.317	0.329	0.293
CG6	0.146	0.201	0.207	0.210	0.179	0.234	0.322	0.331	0.336	0.287
BW12	0.097	0.094	0.104	0.103	0.161	0.198	0.192	0.212	0.210	0.329
WH12	0.112	0.079	0.083	0.097	0.132	0.239	0.168	0.177	0.207	0.281
BL12	0.044	0.044	0.041	0.039	0.043	0.166	0.166	0.155	0.147	0.163
CG12	0.129	0.110	0.163	0.106	0.110	0.220	0.220	0.326	0.212	0.220

BW6: body weight at 6 months; WH6: withers height at 6 months; BL6: body length at 6 months; CG6: chest girth in 6 months; BW12: body weight at 12 months; WH12: withers height at 12 months; BL12: body length at 12 months; CG12: chest girth at 12 months. $r_{GEBV,y}$: the correlation coefficient between GEBV and ***y***; GBLUP: genomic best linear unbiased prediction.

**Table 4:** Correlation test of phenotypic prediction at BW6 and observation at BW30.

	GBLUP	BayesA	BayesB	BayesC	BayesLasso
Pearson correlation coefficient	0.407	0.422	0.403	0.405	0.374
Significance	*p* < 0.001	*p* < 0.001	*p* < 0.001	*p* < 0.001	*p* < 0.001

GBLUP: genomic best linear unbiased prediction.

## Supplementary Materials

The following are available online at https://www.mdpi.com/2076-2615/10/10/1793/s1. The genotypes and phenotypes used in the current study accompany this article at https://doi.org/10.6084/m9.figshare.12496778.v1. Additional file 1 Figure S1: The distribution of minor allele frequency after quality control. Additional file 2 Table S1: Distribution of SNPs on each chromosome before and after quality control.
