# Supplementary material for: Accuracies of Genomic Prediction for Growth Traits at Weaning and Yearling Ages in Yak
Source: Animals (Basel). 2020 Oct 2;10(10):1793. doi: 10.3390/ani10101793 (PMC7650705; doi:10.3390/ani10101793)
Supplement: Supplementary file 1 [file animals-10-01793-s001.zip › TableS1.docx]

Table S1. Distribution of SNPs on each chromosome before and after quality control (QC)

| Chromosome | No. of SNPs before QC | No. of SNPs after QC |
| --- | --- | --- |
| 1 | 46495 | 6396 |
| 2 | 40056 | 4362 |
| 3 | 35579 | 6693 |
| 4 | 34980 | 3871 |
| 5 | 34842 | 6152 |
| 6 | 35519 | 5300 |
| 7 | 33168 | 2964 |
| 8 | 33529 | 2156 |
| 9 | 31060 | 5622 |
| 10 | 30449 | 2663 |
| 11 | 32015 | 2725 |
| 12 | 26127 | 3451 |
| 13 | 23594 | 2746 |
| 14 | 24780 | 1929 |
| 15 | 24755 | 5068 |
| 16 | 24178 | 3317 |
| 17 | 22266 | 5096 |
| 18 | 19386 | 2136 |
| 19 | 18908 | 1675 |
| 20 | 21490 | 3251 |
| 21 | 21175 | 2068 |
| 22 | 18034 | 2600 |
| 23 | 15215 | 5708 |
| 24 | 18620 | 1941 |
| 25 | 12931 | 1967 |
| 26 | 15242 | 1305 |
| 27 | 13152 | 1632 |
| 28 | 13038 | 928 |
| 29 | 14710 | 1594 |
| X | 39367 | 1221 |
