# Supplementary figures and images for: Accuracies of Genomic Prediction for Growth Traits at Weaning and Yearling Ages in Yak
Source: Animals (Basel). 2020 Oct 2;10(10):1793. doi: 10.3390/ani10101793 (PMC7650705; doi:10.3390/ani10101793)

# MAF distribution

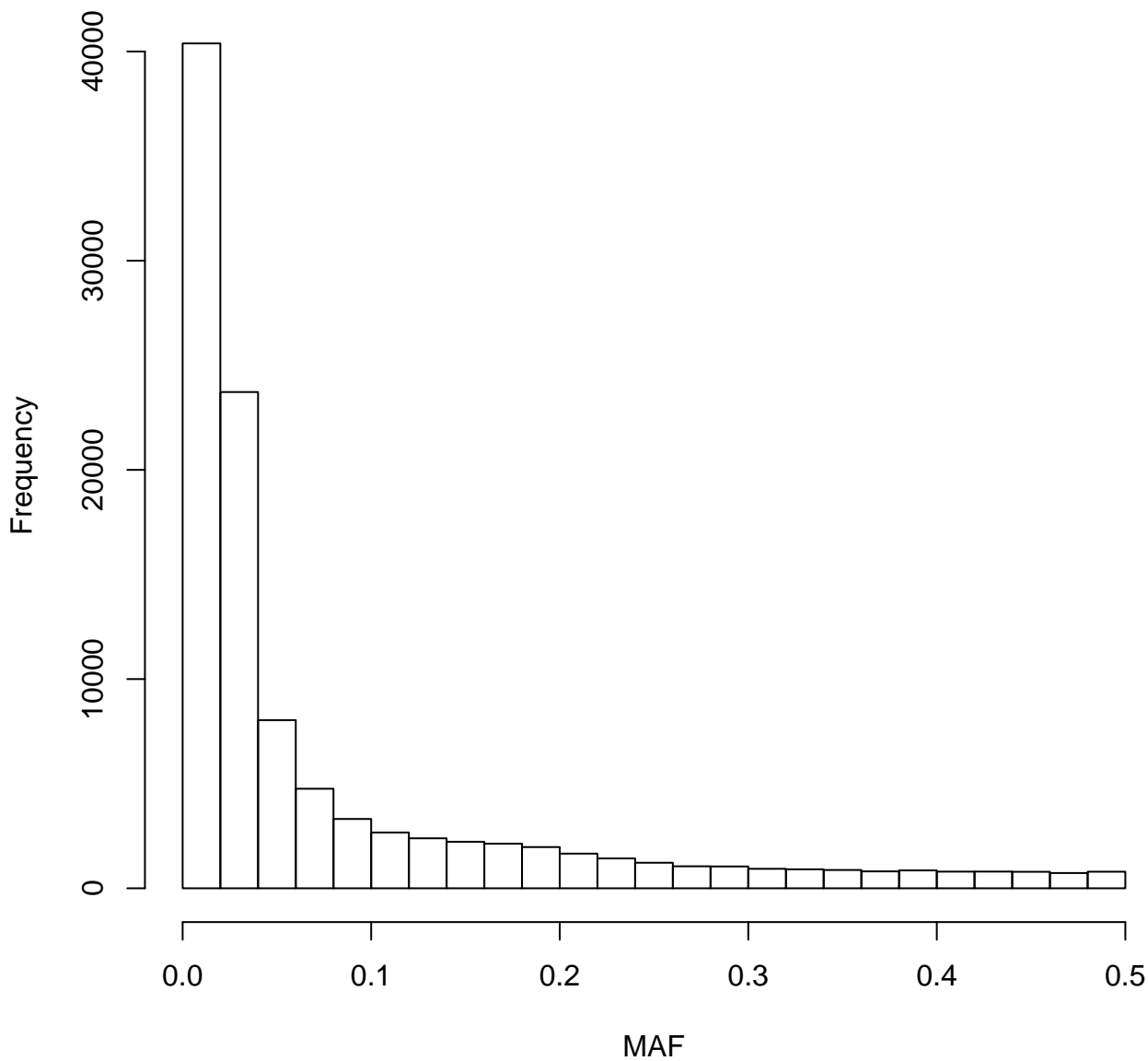

Supplement: Supplementary file 1 [file animals-10-01793-s001.zip › Figure S1.pdf]
